# Association of greenness with incidence of cardiovascular disease in China: Evidence from the China Kadoorie Biobank prospective cohort study with 0.5 million adults

**DOI:** 10.1016/j.eehl.2025.100148

**Published:** 2025-04-24

**Authors:** Xia Meng, Lina Zhang, Ka Hung Chan, Jun Lv, Hubert Lam, Cong Liu, Renjie Chen, Christiana Kartsonaki, Neil Wright, Huaidong Du, Ling Yang, Yiping Chen, Dianjianyi Sun, Pei Pei, Canqing Yu, Haidong Kan, Zhengming Chen, Liming Li, China Kadoorie Biobank Collaborative Group

**Affiliations:** a School of Public Health, Key Lab of Public Health Safety of the Ministry of Education, NHC Key Lab of Health Technology Assessment, Fudan University, Shanghai 200032, China; b School of Public Health, Zhejiang Chinese Medical University, Hangzhou 310053, China; c Clinical Trial Service Unit& Epidemiological Studies Unit, Nuffield Department of Population Health, University of Oxford, Oxford OX3 7LF, UK; d Department of Epidemiology and Biostatistics, School of Public Health, Peking University, Beijing 100191, China; e Peking University Center for Public Health and Epidemic Preparedness & Response, Beijing 100191, China; f Key Laboratory of Epidemiology of Major Diseases (Peking University), Ministry of Education, Beijing 100191, China; g State Key Laboratory of Vascular Homeostasis and Remodeling, Peking University, Beijing 100191, China; h Shanghai Institute of Infectious Disease and Biosecurity, Fudan University, Shanghai 200032, China

**Keywords:** Greenness, Cardiovascular disease, Incidence, Cohort study, Health impact assessment

## Abstract

Prospective evidence on the relationship of greenness with cardiovascular disease (CVD) incidence is limited in low- and middle-income countries. In 512,691 participants of the China Kadoorie Biobank cohort across 10 regions in China, we calculated the levels of greenness exposure based on satellite-retrieved Normalized Difference Vegetation Index (NDVI) data. Annual maximum NDVI (NDVI_max_) values were estimated within 500 ​m and 1000 ​m buffers around the locations for the participants during the follow-up periods. Record linkages to healthcare databases provided incidence data of total CVD, ischemic heart disease and stroke during 2005–2017. Time-varying Cox proportional hazards regression was used to assess the associations between greenness exposure and CVD incidence. After 5.08 million person-years of follow-up, 148,032 incident CVD events were recorded. The overall average level of NDVI_max_ was 0.543 for all participants. We observed significant inverse associations of greenness with the incidence of CVD and its subtypes. Specifically, the hazard ratio for total CVD incidence was 0.976 (95% confidence interval: 0.958, 0.994) per 0.1 increase in NDVI_max_ within a 500 ​m buffer. As the 5 rural regions have achieved the WHO recommended greenness goal values, we compared the greenness levels in the 5 urban regions with the WHO's goal for greenness and found that, on average, 3.81% of total CVD incidence might be averted if the recommended greenness values could be achieved. Exposure to a higher level of greenness was associated with a lower risk of CVD incidence in Chinese adults.

## Highlights

• A significant link was found between greenness and total CVD incidence from 0.5 million adults.
• Protective effects were also observed between greenness and incidence of CVD subtypes.
• 3.81% total CVD incidence might be averted if recommended greenness values could be achieved.

## Graphical abstract

**Figure:**
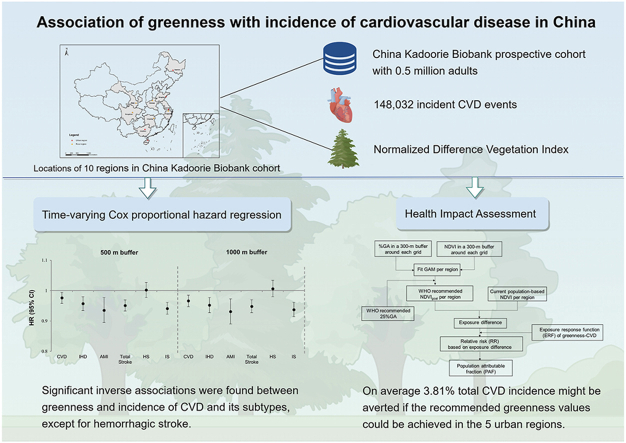

## 1 Introduction

Cardiovascular disease (CVD), primarily ischemic heart disease (IHD) and stroke, is the leading cause of death (19.8 million death in 2022) worldwide, with an increasing burden particularly in low- and middle-income countries (LMICs) in the past three decades [[1], [2], [3]]. Of all potentially modifiable risk factors, greenness (i.e., all vegetated land, such as forests, lawns, parks, gardens, wetlands, and agricultural lands [4]) has drawn growing attention with its potentially wide-ranging health benefits across the life course [[5], [6], [7], [8], [9]], including on CVD risk [6,10,11].

Most existing greenness-CVD studies focused on CVD mortality and found an inverse association with long-term exposure to greenness [10,[12], [13], [14]]. An impact evaluation conducted across 978 European cities found that boosting greenness exposure to the World Health Organization (WHO) recommended level could avert 42,968 premature deaths in 2015, accounting for 2.3% of total natural mortality [15]. Still, CVD mortality is influenced by a greater number of confounding factors between the onset of hospitalization and the occurrence of death compared to CVD incidence [16]. Furthermore, addressing the incidence of CVD could benefit a broad population by effectively mitigating the substantial economic burden. Only a few studies have examined the association of greenness with CVD incidence [6,[17], [18], [19], [20], [21]], which may provide a better insight into possible CVD prevention in relation to greenness than studies on CVD mortality. While these studies hint at a great potential for promoting public health through increasing green space, relevant evidence is scarce in LMICs, where more rapid and drastic changes in the temporal and spatial distribution of green space are observed along with a substantial burden of CVD [22].

China has a substantial CVD burden, and its rapid urbanization over the past three decades has been accompanied by a remarkable rural-to-urban migration, resulting in an uneven spatial distribution of green space exposure [23]. Although some cross-sectional studies on greenness and CVD prevalence have been conducted in China [11,[24], [25], [26]], little prospective cohort evidence exists [27]. Moreover, previous studies were mostly conducted with small sample sizes or in single regions, warranting larger, multi-center studies to ascertain more reliable association estimates [11,14,[24], [25], [26], [27]]. In addition, associations between greenness and the major subtypes of CVD, such as stroke and IHD, have not been systematically investigated and compared in the same study population. Therefore, large prospective cohort studies are urgently needed to understand the role of greenness in CVD (and its subtypes) development in China.

Based on the China Kadoorie Biobank (CKB) prospective cohort study, we aimed to examine whether greenness was associated with the incidence of CVD and its subtypes. We further estimated the potential preventable CVD incidence if the regions in CKB cohort could achieve the WHO recommended greenness values.

## 2 Methods

### 2.1 Study population

**Fig. 1:**
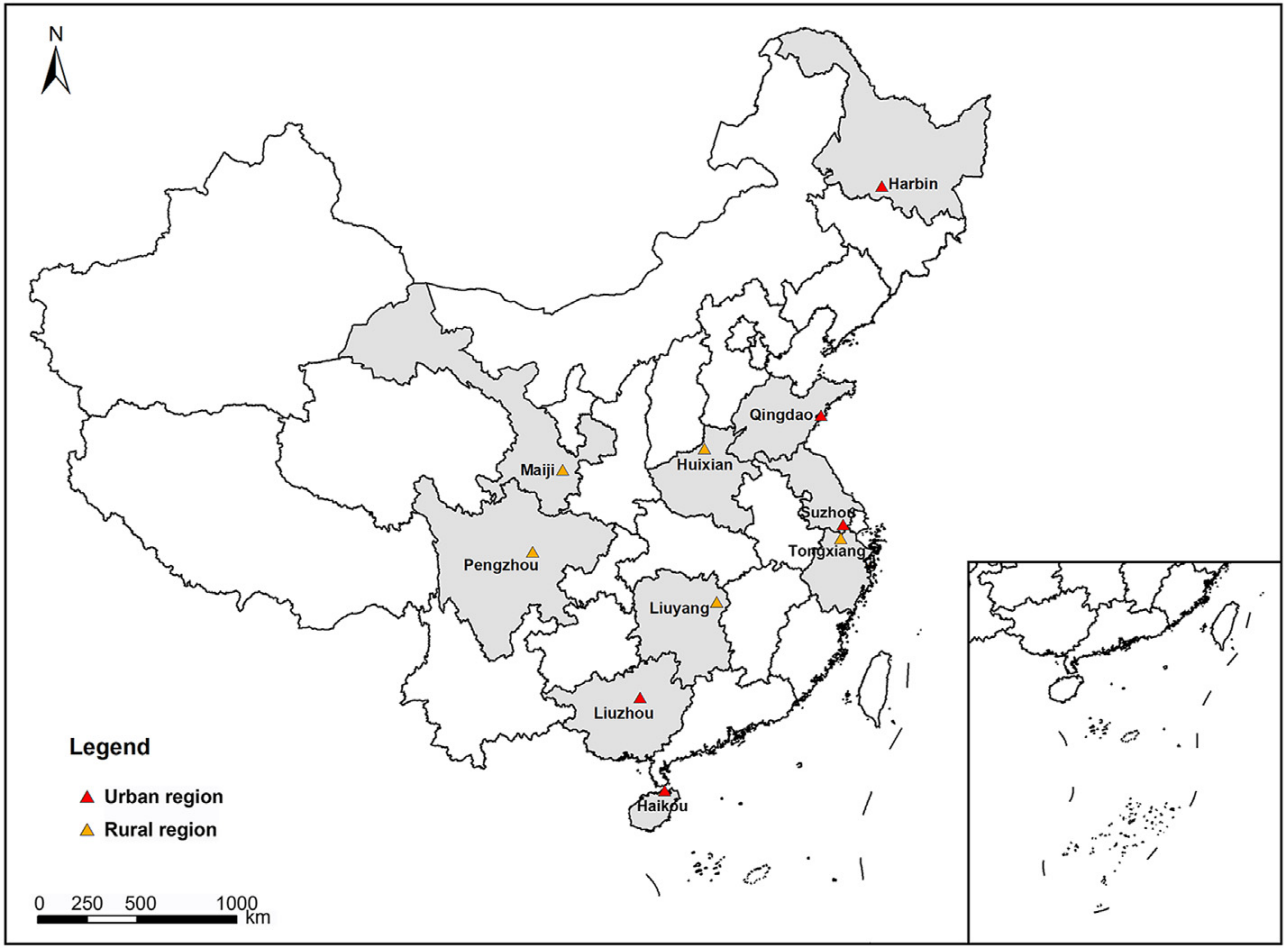
Locations of 10 regions in China Kadoorie Biobank cohort.

The details of the CKB prospective cohort study have been described elsewhere [28,29]. Briefly, 512,691 participants aged 30–79 years were recruited from 10 regions (5 urban and 5 rural areas, Fig. 1) in China during 2004–2008. All participants provided written informed consent. Laptop-based questionnaires were administered by trained interviewers at the local survey clinics set up to enroll residents within ∼1 ​km^2^, assessing information on demographics, socioeconomic status (SES), lifestyle, environmental and other key characteristics of participants. Physical measurements, such as height and weight, were taken by the trained workers.

### 2.2 Assessments of greenness

Exposure to greenness was estimated by vegetation index, which was extracted from retrievals of the Moderate Resolution Imaging Spectroradiometer (MODIS) instrument aboard the Terra satellite. The relevant satellite data with product name MOD13Q1 from January 2005 to December 2017 were downloaded from https://ladsweb.modaps.eosdis.nasa.gov/. The temporal and spatial resolution of the data were 16 days and 250 ​m ​× ​250 ​m, respectively.

Normalized Difference Vegetation Index (NDVI), one of the most commonly used vegetation indices, was used in this study. NDVI ranges from −1 to 1. Higher positive values indicate a higher density of vegetation coverage, values near zero suggest no vegetation or bare land, and negative values represent water or snow. We removed the negative NDVI values to exclude the potential influence of the water body. Satellite data were always affected by meteorological conditions, such as cloud coverage, which may lead to underestimated values of averaged NDVI. Hence, we calculated the maximum NDVI (NDVI_max_) values of the year to represent the maximum coverage of green space as in previous studies [30]. We used 500 ​m and 1000 ​m buffers to estimate the greenness exposure for residential surrounding environment and a 10-min walking distance, respectively [17,31]. Finally, the annual NDVI_max_ level within 500 ​m and 1,000 ​m buffers around the corresponding clinic location recorded at baseline was assigned to the participant each year during the follow-up period. We also used annual mean values of NDVI (NDVI_ann_mean_) as an additional indicator of greenness exposure for sensitivity analysis.

### 2.3 Follow-up and CVD outcomes

All participants were followed up for incident CVD cases through electronic linkages with national health insurance databases and established death and disease (for IHD, stroke, diabetes, and cancers) registries using unique national identification numbers [28]. The national health insurance databases provided comprehensive hospitalization data. According to the International Classification of Diseases, 10th Revision (ICD-10), CVD events were coded as total CVD (I00–I99), IHD (I20–I25), acute myocardial infarction (AMI, I21), total stroke (I60–I61, I63–I64, and I69), ischemic stroke (IS, I63) and hemorrhagic stroke (HS, I61). In this study, we only included the first hospitalization events of CVD during 2005–2017. Participants were followed up until incidence of CVD or were censored if lost to follow-up (<1%), death, or 31 December 2017, whichever came first [32].

### 2.4 Covariates

According to the confounders identified in prior studies on the relationship between greenness and CVD [6,17,20], participants' demographic information, lifestyle, and individual SES collected at baseline were adjusted as potential confounding factors. A directed acyclic graph (Fig. S1) was developed to identify minimally sufficient adjustments of these covariates [33]. Basic information included sex (male, female), age, body mass index (BMI, <18.5, 18.5–24.9, ≥25 ​kg/m^2^); individual SES variables included education levels (never, primary school, high school, college/higher), household income (<10,000, 10,000–19,999, 20,000–34,999, ≥35,000 yuan/year); lifestyle factors included smoking (never, occasionally, ex-regular, current-regular), secondhand smoke (never, former, present), alcohol drinking (never, ex-regular, occasionally, monthly, weekly), fruits, vegetables, fish and meat intake (daily, 4–6 days/week, 1–3 days/week, monthly, never/rarely), cooking fuels (clean, unclean to clean, unclean, never cooking, others), heating fuels (clean, unclean to clean, unclean, never heating, others), physical activity [metabolic equivalent of task (MET, hours/day)], self-rated health (excellent, good, fair, poor). The daily mean fine particulate matter (PM_2.5_) concentrations and daily maximum 8-h ozone (O_3_) concentrations were estimated at a 1 ​km ​× ​1 ​km resolution in mainland China following the methodologies described in previous studies [34,35]. Furthermore, annual concentrations of PM_2.5_ and O_3_ were assigned to each participant during the follow-up period based on the clinic location recorded at baseline, which was calculated as potential confounders in sensitivity analyses.

### 2.5 Statistical analyses

#### 2.5.1 Effects estimation

We used time-varying Cox proportional hazards regression models to assess the associations between greenness and CVD incidence. The annual greenness exposure for each year from baseline to the year of incident CVD was included in the Cox proportional hazard regression models as a time-varying variable, as the levels of greenness varied each year, and the trend might be inconsistent in different regions [36]. Covariates involved in the fully adjusted model included strata of regions and clusters of clinics, and adjustment for age, sex, smoking, secondhand smoke, alcohol drinking, education, income, BMI, MET, fresh fruits, fresh vegetables, fish, meat, cooking fuels, heating fuels, and self-rated health. The hazard ratios (HRs) and 95% confidence intervals (CIs) of CVD incidence were calculated per 0.1 change of NDVI_max_. The exposure-response relationships between greenness (NDVI_max_) and incidence of CVD and its subtypes were analyzed using natural spline functions within the fully adjusted model [11,32].

Subgroup analyses were conducted by age (<65, ≥65 years), sex (male, female), education(low: junior high school or below, high: senior high school or higher), income (low: <20,000 yuan/year, high: ≥20,000 yuan/year), physical activity (MET, low: <13 ​h/day, middle: 13–25.9 ​h/day, high: ≥26 ​h/day), BMI (<18.5, 18.5–24.9, ≥25 ​kg/m^2^), self-rated health (poor, non-poor), PM_2.5_ (low: 19.60–53.54 ​μg/m^3^, high: 53.55–82.41 ​μg/m^3^) and O_3_ (low: 28.91–55.07 ​ppb, high: 55.08–76.95 ​ppb) to investigate the potential effect modification. Then, we tested whether the differences in risk estimates among subgroups of these potential modifiers were statistically significant [37,38]. The specific formula employed was detailed in the Supplementary Information (SI) Methods. Additionally, mediation analyses were conducted to investigate potential mediating roles of PM_2.5_ and O_3_.

For sensitivity analyses, firstly, we used NDVI_ann_mean_ in the exposure assessment of greenness. Secondly, we excluded the participants with self-reported CVD (i.e., hypertension, stroke, or IHD) at baseline. To minimize potential reverse causation, participants with a follow-up period of less than two years were also excluded. Thirdly, BMI subcategories were redefined according to the criteria for overweight in the Chinese population [39,40]. Fourthly, we further adjusted for PM_2.5_ and O_3_ as potential confounders.

#### 2.5.2 Quantitative assessments on the preventable CVD incidence with respect to the WHO recommended levels

**Fig. 2:**
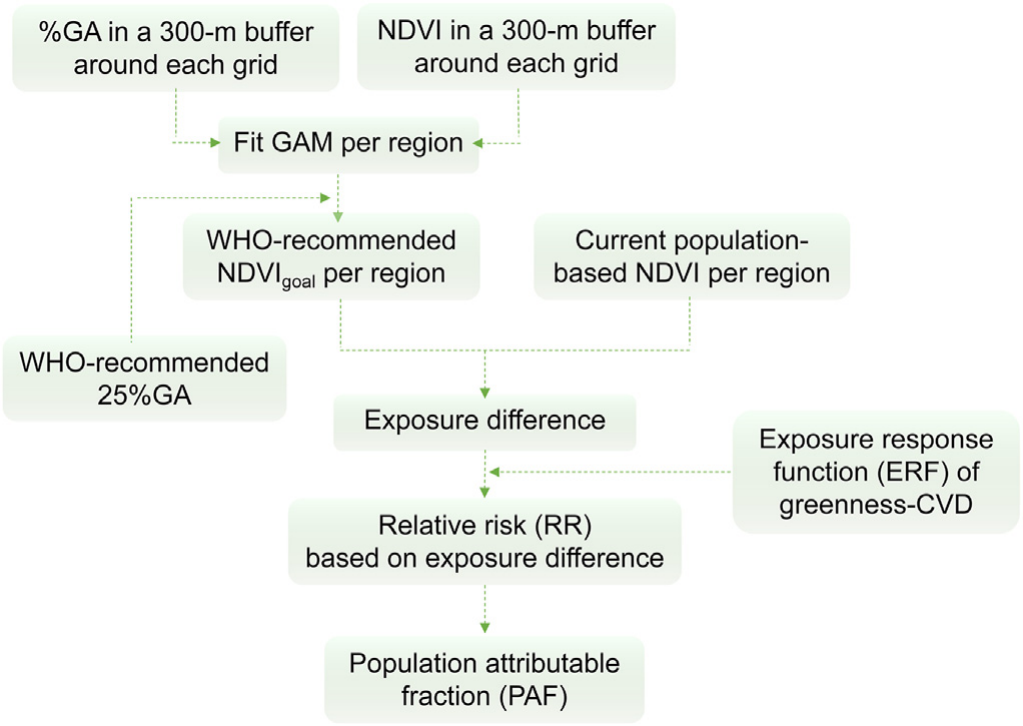
The framework of calculating preventable proportion of CVD incidence attributable to greenness exposure in this study.

We conducted a health impact assessment (HIA) at city scale following the methodology of the Urban and Transport Planning Health Impact Assessment [15,41], with the procedures summarized in Fig. 2. Based on the WHO recommended universally accessible green space (at least 0.5 ​ha within a linear distance of 300 ​m), studies found that about 25% green area (25%GA) was correspondingly needed per unit area [15,42,43]. Greenness was mostly estimated by NDVI for effect estimates in epidemiological studies; hence, we established the relationship between NDVI and the percentage of green space area (%GA) using a generalized additive model (GAM) in this study following the method proposed in previous study, to decide the corresponding value of NDVI (NDVI_goal_) for 25%GA in each region in CKB. Second, we evaluated the population-weighted NDVI level in 2020 in each CKB region to assess whether the averaged population exposure level to greenness met the WHO recommendation (NDVI_goal_) and the proportion of population living below NDVI_goal_. Third, based on the effect estimates from this study, we further calculated the preventable CVD incidence in a hypothetical scenario where these regions in CKB could meet NDVI_goal_. The details of methodology in this section are provided in SI Methods.

We conducted statistical analyses with R (version 4.2.2), including the packages of survival, regmedint and forestplot. Two-sided *p* values ​< ​0.05 indicated statistical significance.

## 3 Results

### 3.1 Descriptive statistics

This study spanned from January 2005 to December 2017, encompassing 5.08 million person-years of follow-up with a median follow-up duration of 11.2 years. Specifically, 148,032 incident cases of CVD were recorded among the 512,691 participants, including 50,323 IHD, 4604 AMI, 57,224 total stroke, 50,175 IS, and 7685 HS cases. Compared to the non-CVD group, participants who developed CVD tended to be older (CVD vs. non-CVD: 57.20 vs. 49.92 years old), overweight (CVD vs. non-CVD: 39.4% vs. 30.3% with BMI ≥ 25 ​kg/m^2^), and to have less physical activity (CVD vs. non-CVD: 17.90 vs. 22.36 ​h/day MET) (Table 1). The mean (SD) NDVI_max_ was 0.543 (0.188) during 2005–2017 for all participants in the cohort, where the mean (SD) NDVI_max_ in urban and rural regions in CKB were 0.377 (0.124) and 0.674 (0.111), respectively. Participants with CVD lived in a less green environment (NDVI_max_: 0.531) than those without CVD (NDVI_max_: 0.546) overall (Table 1).

**Table 1:** Descriptive characteristics of participants in China Kadoorie Biobank at baseline and averaged exposure levels of environmental factors for participants during baseline and follow-up periods.

Variables	CVD	Non-CVD	*P*
	(n ​= ​148,032)	(n ​= ​364,659)	
**Age** (years, Mean ​± ​SD)	57.20 ​± ​10.06	49.92 ​± ​10.20	<0.001
**Sex** (n, %)			
Male	60,203 (40.7)	149,992 (41.1)	0.002
Female	87,829 (59.3)	214,667 (58.9)	
**BMI** (n, %)			
<18.5 ​kg/m^2^	6193 (4.2)	16,167 (4.4)	<0.001
18.5–24.9 ​kg/m^2^	83,572 (56.5)	237,851 (65.2)	
≥25 ​kg/m^2^	58,267 (39.4)	110,639 (30.3)	
**Education** (n, %)			
No formal education	33,475 (22.6)	61,695 (16.9)	<0.001
Primary school	51,522 (34.8)	113,651 (31.2)	
High school	53,983 (36.5)	168,392 (46.2)	
College or higher	9052 (6.1)	20,921 (5.7)	
**Income** (n, %)			
<10,000 yuan/year	42,406 (28.6)	102,316 (28.1)	<0.001
10,000–19,999 yuan/year	44,141 (29.8)	104,804 (28.7)	
20,000–34,999 yuan/year	36,235 (24.5)	90,464 (24.8)	
≥35,000 yuan/year	25,250 (17.1)	67,075 (18.4)	
**Smoking** (n, %)			
Never	90,951 (61.4)	226,520 (62.1)	<0.001
Occasionally	7760 (5.2)	21,384 (5.9)	
Ex-regular	12,090 (8.2)	18,469 (5.1)	
Current-regular	37,231 (25.2)	98,286 (27.0)	
**Secondhand smoke** (n, %)			
Never	37,432 (25.3)	88,236 (24.2)	<0.001
Former^a^	46,195 (31.2)	114,281 (31.3)	
Present	64,405 (43.5)	162,142 (44.5)	
**Alcohol drinking** (n, %)			
Never	71,133 (48.1)	163,960 (45.0)	<0.001
Ex-regular	3985 (2.7)	5268 (1.4)	
Occasionally	43,773 (29.6)	119,350 (32.7)	
Monthly	8407 (5.7)	20,674 (5.7)	
Weekly	20,734 (14.0)	55,407 (15.2)	
**Physical activity** (MET, hours/day, Mean ​± ​SD)	17.90 ​± ​12.99	22.36 ​± ​14.01	<0.001
**Fruits** (n, %)			
Daily	29,015 (19.6)	67,561 (18.5)	<0.001
4−6 days/week	12,148 (8.2)	35,804 (9.8)	
1−3 days/week	44,095 (29.8)	117,190 (32.1)	
Monthly	51,801 (35.0)	122,314 (33.5)	
Never/Rarely	10,973 (7.4)	21,790 (6.0)	
**Vegetables** (n, %)			
Daily	140,570 (95.0)	345,346 (94.7)	<0.001
4−6 days/week	5109 (3.5)	12,950 (3.6)	
1−3 days/week	1961 (1.3)	5228 (1.4)	
Monthly	348 (0.2)	1052 (0.3)	
Never/Rarely	44 (0.0)	83 (0.0)	
**Fish** (n, %)			
Daily	3524 (2.4)	10,631 (2.9)	<0.001
4−6 days/week	7614 (5.1)	23,789 (6.5)	
1−3 days/week	53,776 (36.3)	140,738 (38.6)	
Monthly	36,229 (24.5)	72,477 (19.9)	
Never/Rarely	46,889 (31.7)	117,024 (32.1)	
**Meat** (n, %)			
Daily	41,941 (28.3)	108,070 (29.6)	<0.001
4−6 days/week	25,353 (17.1)	66,447 (18.2)	
1−3 days/week	52,805 (35.7)	129,354 (35.5)	
Monthly	19,835 (13.4)	44,384 (12.2)	
Never/Rarely	8098 (5.5)	16,404 (4.5)	
**Cooking fuels** (n, %)			
Clean	21,483 (14.7)	63,921 (17.8)	<0.001
Unclean to clean^b^	29,806 (20.4)	65,979 (18.4)	
Unclean	51,965 (35.6)	120,981 (33.7)	
Never cooking	35,963 (24.6)	90,834 (25.3)	
Others	6864 (4.7)	17,172 (4.8)	
**Heating fuels** (n, %)			
Clean	12,309 (8.3)	31,825 (8.7)	<0.001
Unclean to clean^b^	22,935 (15.5)	34,901 (9.6)	
Unclean	55,398 (37.4)	130,410 (35.8)	
Never heating	50,098 (33.8)	149,144 (40.9)	
Others	7291 (4.9)	18,379 (5.0)	
**Self-rated health** (n, %)			
Excellent	21,375 (14.4)	69,017 (18.9)	
Good	35,690 (24.1)	108,513 (29.8)	<0.001
Fair	70,575 (47.7)	154,439 (42.4)	
Poor	20,392 (13.8)	32,690 (9.0)	
**NDVI_max_** (Mean ​± ​SD)	0.531 ​± ​0.200	0.546 ​± ​0.186	<0.001
**NDVI_ann_mean_** (Mean ​± ​SD)	0.312 ​± ​0.130	0.329 ​± ​0.121	<0.001
**PM_2.5_** (μg/m^3^, Mean ​± ​SD)	53.341 ​± ​11.474	52.409 ​± ​11.721	<0.001
**O_3_** (ppb, Mean ​± ​SD)	53.565 ​± ​8.552	54.386 ​± ​8.572	<0.001

a Refers to individuals previously exposed to secondhand smoke.

b Refers to a shift from using unclean fuels, such as coal and wood, to clean fuels, such as gas and electricity. CVD, cardiovascular disease; BMI, body mass index; MET, metabolic equivalent of task; NDVI, Normalized Difference Vegetation Index; PM_2.5_, fine particulate matter; O_3_, ozone; NDVI_max_, the overall average level of NDVI_max_; NDVI_ann_mean_, the overall average level of NDVI_annual_.

### 3.2 Associations between greenness and CVD incidence

The exposure-response curves showed a decreasing trend for CVD, IHD, AMI, total stroke, and IS (Fig. S2A-E), except for HS with notably wide CIs (Fig. S2F). Table 2 shows that higher greenness was associated with lower CVD incidence risks. In particular, in a 500 ​m buffer, greenness was associated with a lower risk of total CVD incidence [HR 0.976 (95% CI: 0.958, 0.994)] per 0.1 increase in NDVI_max_. For CVD subtypes, greenness was significantly associated with lower risks of incident IHD [HR 0.957 (95% CI: 0.934, 0.980)], AMI [HR 0.935 (95% CI:0.895, 0.977)], stroke [HR 0.951 (95% CI: 0.933, 0.969)], IS [HR 0.941 (95% CI: 0.922, 0.961)] per 0.1 increase in NDVI_max_, respectively, but not with HS [HR 1.002 (95% CI: 0.977, 1.027)]. The associations with NDVI_max_ in a 1000 ​m buffer were slightly stronger and quite largely consistent with those based on NDVI_max_ with a 500 ​m buffer.

**Table 2:** Hazard ratios (HRs) and 95% confidence intervals (95% CI) of CVD incidence per 0.1 increment in NDVI_max_ within 500 ​m and 1000 ​m buffers.

Outcomes	500 ​m buffer	1000 ​m buffer
Cardiovascular disease (CVD)	0.976 (0.958, 0.994)	0.966 (0.947, 0.985)
Ischaemic heart disease (IHD)	0.957 (0.934, 0.980)	0.952 (0.927, 0.977)
Acute myocardial infarction (AMI)	0.935 (0.895, 0.977)	0.931 (0.890, 0.973)
Total Stroke	0.951 (0.933, 0.969)	0.948 (0.927, 0.970)
Hemorrhagic stroke (HS)	1.002 (0.977, 1.027)	1.006 (0.980, 1.034)
Ischaemic stroke (IS)	0.941 (0.922, 0.961)	0.937 (0.914, 0.961)

The fully adjusted model included strata of regions and clusters of clinics, and adjustment for age, sex, smoking, secondhand smoke, alcohol drinking, education, income, BMI, MET, fresh fruits, fresh vegetables, fish, meat, cooking fuels, heating fuels and self-rated health.

### 3.3 Stratified analyses

**Fig. 3:**
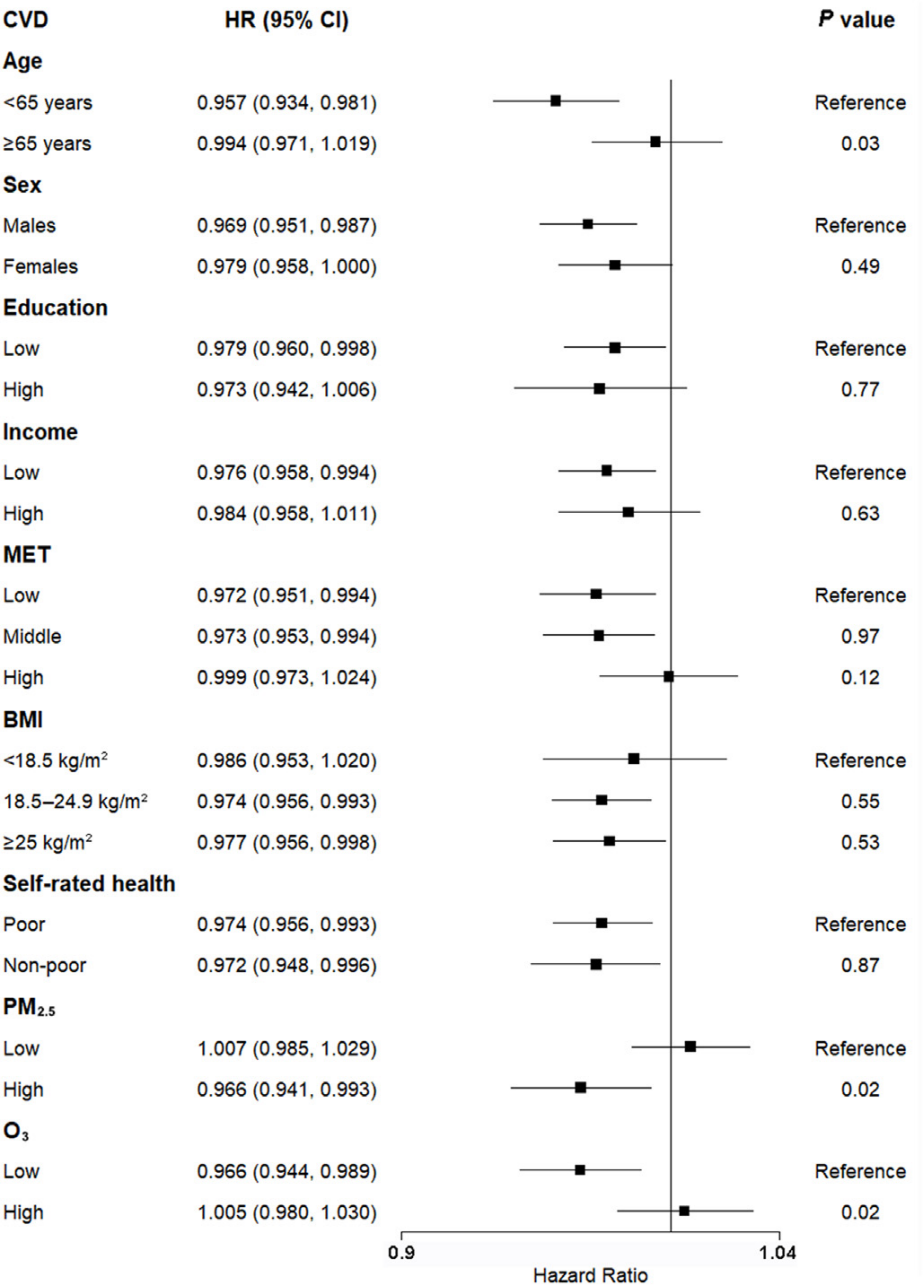
Stratified analyses for associations between NDVI_max_ and total cardiovascular disease (CVD) incidence. Statistical significance (*P* value) was calculated between the target and reference subgroups.

Effect estimates of greenness on CVD incidence were largely consistent between subgroups, except for age and air pollutants (Fig. 3). In particular, the association appeared to be predominantly in younger (<65 years) [HR 0.957 (95% CI: 0.934, 0.981)] but not in older (≥65 years) [HR 0.994 (95% CI: 0.971, 1.019)] participants; the protective effect of greenness on CVD incidence was statistically significant only in regions with high concentrations of PM_2.5_ [HR 0.966 (95% CI: 0.941, 0.993)], whereas the results were reversed for O_3_. Moreover, significant associations were observed among participants with lower levels of education and income, whereas no significant associations were found in the corresponding higher education and income subgroups. Furthermore, the association was statistically significant in the low and middle levels of physical activity subgroups, but not in the high-level group. This discrepancy may be attributed to SES and health characteristics of participants across different levels of MET, as detailed in Table S1.

### 3.4 Sensitivity analyses

The results remained robust across the sensitivity analyses. First, when using NDVI_ann_mean_ in exposure assessment, the effect estimates were similar to the results in the main analysis (Table S2). Second, we excluded the participants with self-reported CVD at baseline, and the results were still robust (Table S3). The results remained robust, albeit slightly weaker, when participants with less than two years of follow-up were excluded, except for CVD within a 500 ​m buffer (Table S4). Third, there was little change after redefining the subcategories of BMI in Chinese classification (Table S5). Fourth, with further adjustment of potential confounders by PM_2.5_ and O_3_, the results were almost the same (Table S6).

### 3.5 Mediation analyses of PM_2.5_ and O_3_

We examined the mediating roles of PM_2.5_ and O_3_ in the relationship between greenness and incidence of CVD and its subtypes, except for the non-significant association between greenness and HS (Table S7). Our results indicated that O_3_ significantly mediated 13.1%–52.8% of the associations between greenness and incidence of CVD and its subtypes (Table S7). In contrast, PM_2.5_ did not significantly mediate, or only minimally mediated, the relationship between greenness and incidence of CVD and its subtypes.

### 3.6 Preventable CVD incidence

Table 3 shows, on average, 60% of the residents in the five CKB urban areas (Harbin, Qingdao, Liuzhou, Haikou, Suzhou) and 24% of those in the five rural areas were living with greenness levels below the WHO recommendation. Among the 10 CKB regions, the population-weighted average greenness levels in the five CKB urban areas in 2020 did not achieve their corresponding NDVI_goal_ values, but the five rural CKB areas surpassed their corresponding NDVI_goal_. Therefore, we estimated the preventable incidence in the five CKB urban areas. We found that 2.24%–7.39% (3.81% on average) of the CVD incidence might be prevented in the five urban areas, if they could achieve the WHO recommended greenness level, with the highest health benefit in the urban area in Harbin and the lowest in the site in Haikou (Table 3).

**Table 3:** Preventable CVD incidence if the population-weighted NDVI level achieved the WHO recommendation per region.

Area	Region	NDVI_goal_	Current NDVI level	Proportion of Population living below NDVI_goal_	Preventable PAF (%) and 95% CI
Urban area	Harbin	0.647	0.328	80.14%	7.39 (1.74, 12.72)
	Qingdao	0.525	0.372	70.56%	3.62 (0.84, 6.32)
	Liuzhou	0.499	0.385	52.65%	2.70 (0.62, 4.74)
	Haikou	0.548	0.454	44.39%	2.24 (0.51, 3.93)
	Suzhou	0.554	0.423	51.34%	3.11 (0.72, 5.44)
Rural area	Pengzhou	0.521	0.624	21.85%	/
	Maiji	0.521	0.611	19.51%	/
	Tongxiang	0.469	0.508	38.08%	/
	Liuyang	0.541	0.695	13.30%	/
	Huixian	0.617	0.678	28.37%	/

“/” represents that the population-weighted NDVI has achieved the NDVI_goal_. PAF, population attributable fraction.

## 4 Discussion

In this large prospective study of 512,691 Chinese adults, we found that higher greenness was associated with lower CVD incidence risk, and on average 3.81% of CVD incidence could be prevented by improving greenness level in the CKB urban regions. To the best of our knowledge, this is the first cohort study in LMICs to assess the association of greenness with CVD incidence and the potential health benefits gained with greenness increments according to WHO recommendations.

The magnitude of the inverse association of greenness with CVD incidence in this study is consistent with previous studies, although they were mostly conducted in high-income countries [6,[17], [18], [19], [20], [21],^27^,^44^,^45^]. For instance, in Rome, a cohort study with 1,254,030 participants found an HR of 0.976 (95% CI: 0.960, 0.993) per interquartile range (IQR, 0.1) increase in NDVI for stroke incidence [17]. Similar results were obtained when greenness exposure of the leaf area index was applied. Nevertheless, this administrative cohort in Rome was characterized by a lack of individual-level confounders, such as lifestyles. In Ontario, Canada, a cohort study with 1,290,288 participants found an HR of 0.93 (95% CI: 0.91, 0.96) and 0.94 (95% CI: 0.93, 0.96) for AMI and heart failure incidence associated with per IQR (0.17) increment in NDVI, respectively [6]. Although the protective effects in the two studies were comparable in magnitude to ours, they only covered limited CVD subtypes. In comparison, a Korean study, like ours, covered both total CVD events and major subtypes but showed stronger protective effects, possibly due to different greenness exposure assessment [20]. Concretely, in the prospective study with 351,409 participants in seven cities, participants in the highest quartile of greenness exposure (proportion of green space in each district, 4.47%) observed an HR of 0.85 (95% CI: 0.81, 0.89) for total CVD events (death or at least 2 days of hospitalization) compared to those in the lowest quantile of greenness (0.18%) [20]. Specifically, they found HRs of 0.83 (95% CI: 0.78, 0.89), 0.77 (95% CI: 0.68, 0.88), 0.87 (95% CI: 0.82, 0.93), 0.86 (95% CI: 0.80, 0.94) and 0.98 (95% CI: 0.86, 1.12) for coronary heart disease, AMI, total stroke, IS and HS events in the highest quantile greenness, compared to those in the lowest one, respectively [20]. Most previous studies found an inverse association between greenness and CVD incidence, while one cross-sectional study observed positive (hazardous) associations of greenness with CHD and stroke prevalence in Atlanta [46]. Differences in effect estimates from multiple studies may be due to different study regions, designs, and methods of greenness exposure assessment.

Prospective cohort evidence in LMICs is scarce, with the exception of one study conducted within a district in China [27]. To date, only a few cross-sectional studies (mostly confined to a single city or region) have examined the association between greenness and CVD prevalence in LMICs, such as in China and Iran [11,24,26,47], and they found hints of potential benefits, but the lack of temporality and generalizability limited their ability of inference. Based on a prospective cohort of 0.5 million participants recruited across 10 geographically diverse regions in China, our study captured a wide range of greenness exposure levels in a large study population that has undergone rapid urbanization and changes in greenness exposure in recent decades. The prospective nature, large case number, population and geographical diversity of our study enabled an assessment of temporality and generation of more robust and generalizable effect estimates. Our results contribute to the growing body of evidence supporting the protective effects of greenness on CVD incidence in LMICs.

Notably, in our subgroup analysis, we observed a stronger association of greenness with total CVD incidence in younger (<65 years) than older (≥65 years) participants, corroborating several previous studies [17,18]. One postulated reason concerns the higher background CVD risk in the elderly, which might have masked the potential benefits of greenness [10]. Furthermore, our study demonstrated that participants with lower SES, characterized by factors such as education and income, and those exposed to higher levels of PM_2.5_ concentrations were more likely to derive benefits. This could also explain why the protective association was not observed in the high-level physical activity group in this study. This finding aligned with previous research, which suggested that individuals living in lower SES conditions and polluted environments were more susceptible to the protective effects of greenness [48,49]. Current evidence on susceptible population is limited and still needs to be explored in depth, especially through cohort studies.

For the first time in any LMICs, we found that if the WHO recommended greenness level were achieved in the five urban areas of CKB, on average, 3.81% (2.24%–7.39%) of CVD incidence might be averted. These figures are comparable to the preventable mortality attributed to the improvement of greenness in Europe and North America [15,41,[50], [51], [52]]. For instance, one of the largest studies of its kind involved 978 cities in Europe, where half of the population lived below the WHO recommended greenness level, found 0.2%–5.5% total preventable natural-cause mortality by achieving the WHO recommendation [15]. Another health impact assessment study estimated that 2.9% of the total mortality can be prevented annually if the tree canopy cover increased from 20.3% in 2014 to 30% in 2025 in Philadelphia (PA, USA) [51]. Although the potential preventable CVD incidence attributed to higher greenness is relatively small in proportion, the substantial CVD burden in China and worldwide means major absolute health gains. Compared to other health promotion strategies such as health education or increased investment in healthcare systems, increasing greenness via urban planning could establish passive benefits not only to the public but also to planetary health.

The mechanisms of the benefit of greenness on CVD risk remain unclear. Several potential pathways have been proposed. First, human beings have a native connection with nature, which may reduce psycho-physiological stress [53,54], an essential risk factor for CVD [55]. Second, some studies suggested that greenness may promote human health through lowering air pollution and promoting physical activity or social engagement and cohesion [56,57], which are influential factors for CVD. Furthermore, the studies tested potential mediation effects of air pollutants, physical activity or social engagement on the health effects of greenness, which suggested that small mediation effects of air pollutants or physical activity but larger mediation effects of social engagement [58,59]. Lower social cohesion or social support might be associated with increased risks of CVD through activating the hypothalamic-pituitary-adrenal (HPA) axis or raising inflammatory cytokine levels [60]. Our findings suggested that air pollutants did not significantly mediate or mediate in small part in the relationship between greenness and CVD. Existing evidence on the mediating effect of air pollutants remained inconclusive [56]. Additionally, these factors were also assessed as potential effect modifiers [14,58,61]. Overall, more studies are still required to understand the underlying mechanisms linking greenness to CVD.

There are several limitations in the present study. First, the characteristics of green space (e.g. tidiness, safety, plantation species) could not be classified by the satellite-based NDVI index. It was challenging to ascertain detailed green space characteristics in this large-scale multicenter study, whereas using NDVI enabled quantitative assessment of the association between greenness and CVD with a uniform parameter adopted in the existing evaluation on greenness. Further methodological development is needed to facilitate detailed characterization of green space using multi-dimensional and multi-scale parameters. Second, as in most previous studies, the time participants spent in green space was not available, which may have led to exposure misclassification. However, with the large sample size and population diversity, the between participants variability of time spent in green space is likely to be accounted for as random error and reflected in the CIs of the HR estimates. Third, we did not account for participant migration during the follow-up period for greenness assessment. Although the CKB cohort study conducts repeated surveys every five years following the baseline survey, it only involves a random selection of 5% of participants for comprehensive re-examination [29]. Consequently, changes in residential addresses are not available for all participants. Last, while we could not obtain the precise timing of CVD incidence, which presents a challenge, the primary aim of this study was to establish the association between greenness and CVD incidence rather than the temporality of CVD development. Despite this, the incidence endpoints for CVD and its subtypes in the CKB cohort provided a high diagnostic quality for examining the relationship with greenness in this study. Future studies may be enhanced by obtaining individual activity information using wearable devices to more accurately estimate exposure level to greenness.

## 5 Conclusion

Based on a large population cohort covering 10 diverse regions of China, we provided important evidence on the health benefits of greenness on CVD incidence in rapidly developing LIMCs settings. Our findings lend further support to the public health value of increasing green space via urban planning.

## CRediT authorship contribution statement

**Xia Meng:** Writing – review & editing, Writing – original draft, Methodology, Formal analysis. **Lina Zhang:** Writing – review & editing, Writing – original draft, Methodology, Formal analysis. **Ka Hung Chan:** Writing – review & editing, Writing – original draft, Methodology, Formal analysis. **Jun Lv:** Writing – original draft, Methodology, Formal analysis. **Hubert Lam:** Writing – review & editing, Formal analysis. **Cong Liu:** Writing – review & editing, Formal analysis. **Renjie Chen:** Writing – review & editing, Formal analysis. **Christiana Kartsonaki:** Writing – review & editing, Formal analysis. **Neil Wright:** Writing – review & editing, Formal analysis. **Huaidong Du:** Writing – review & editing, Formal analysis. **Ling Yang:** Writing – review & editing, Formal analysis. **Yiping Chen:** Writing – review & editing, Formal analysis. **Dianjianyi Sun:** Writing – review & editing, Methodology, Data curation. **Pei Pei:** Writing – review & editing, Methodology, Data curation. **Canqing Yu:** Writing – review & editing, Methodology, Data curation. **Haidong Kan:** Writing – review & editing, Supervision, Funding acquisition, Conceptualization. **Zhengming Chen:** Writing – review & editing, Supervision, Funding acquisition, Conceptualization. **Liming Li:** Writing – review & editing, Supervision, Funding acquisition, Conceptualization.

## Declaration of competing interests

The authors declare no competing interests.
