# Supplementary material for: Association of greenness with incidence of cardiovascular disease in China: Evidence from the China Kadoorie Biobank prospective cohort study with 0.5 million adults
Source: Eco Environ Health. 2025 Apr 24;4(2):100148. doi: 10.1016/j.eehl.2025.100148 (PMC12141932; doi:10.1016/j.eehl.2025.100148)

**Supplementary Information**

**Association of greenness with incidence of cardiovascular disease in China: Evidence from the** **China Kadoorie Biobank prospective cohort study with 0.5 million adults**

Xia Meng^a,1^, Lina Zhang^b, 1^, Ka Hung Chan^c,1^, Jun Lv^d,e,f,g,1^, Hubert Lam^c^, Cong Liu^a^, Renjie Chen^a^, Christiana Kartsonaki^c^, Neil Wright^c^, Huaidong Du^c^, Ling Yang^c^, Yiping Chen^c^, Dianjianyi Sun^d,e,f^, Pei Pei^e^, Canqing Yu^d,e,f^, Haidong Kan^a,h,^*, Zhengming Chen^c,^*, Liming Li^d,e,f,^*, China Kadoorie Biobank Collaborative Group

^a^ School of Public Health, Key Lab of Public Health Safety of the Ministry of Education, NHC Key Lab of Health Technology Assessment, Fudan University, Shanghai 200032, China

^b^ School of Public Health, Zhejiang Chinese Medical University, Hangzhou 310053, China

^c^ Clinical Trial Service Unit& Epidemiological Studies Unit, Nuffield Department of Population Health, University of Oxford, Oxford OX3 7LF, UK

^d^ Department of Epidemiology and Biostatistics, School of Public Health, Peking University, Beijing 100191, China

^e^ Peking University Center for Public Health and Epidemic Preparedness & Response, Beijing 100191, China

^f^ Key Laboratory of Epidemiology of Major Diseases (Peking University), Ministry of Education, Beijing 100191, China

^g^ State Key Laboratory of Vascular Homeostasis and Remodeling, Peking University, Beijing 100191, China

^h^ Shanghai Institute of Infectious Disease and Biosecurity, Fudan University, Shanghai 200032, China

^1^ These authors contributed equally to this work.

* Corresponding authors.

E-mail: [lmlee@vip.163.com](mailto:lmlee@vip.163.com) (L. Li); [zhengming.chen@ndph.ox.ac.uk](mailto:zhengming.chen@ndph.ox.ac.uk) (Z. Chen); [kanh@fudan.edu.cn](mailto:kanh@fudan.edu.cn) (H. Kan)

**Methods**

The formula for statistical significance of differences among subgroup analyses.

Quantitative assessments on the preventable CVD incidence if met the WHO recommended levels.

**Table S1.** SES and health characteristics of participants in China Kadoorie Biobank at baseline, stratified by subgroups of MET

**Table S2.** Hazard ratios (HRs) and 95% confidence intervals (95% CI) of CVD incidence per 0.1 increment in NDVI_ann-mean_ within 500 m and 1000 m buffers

**Table S3.** Hazard ratios (HRs) and 95% confidence intervals (95% CI) of CVD incidence per 0.1 increment in NDVI_max_ within 500 m and 1000 m buffers by excluding the participants with self-reported CVD at baseline

**Table S4.** Hazard ratios (HRs) and 95% confidence intervals (95% CI) of CVD incidence per 0.1 increment in NDVI_max_ within 500 m and 1000 m buffers by excluding the participants with follow-up time less than 2 years

**Table S5.** Hazard ratios (HRs) and 95% confidence intervals (95% CI) of CVD incidence per 0.1 increment in NDVI_max_ within 500 m and 1000 m buffers for the redefinition of the subgroups of BMI

**Table S6.** Hazard ratios (HRs) and 95% confidence intervals (95% CI) of CVD incidence per 0.1 increment in NDVI_max_ within 500 m and 1000 m buffers after further adjustment of PM_2.5_ and O_3_

**Table S7.** The proportion of mediation of associations between greenness and CVD incidence within a 500 m buffer, by air pollutants

**Fig. S1.** Directed acyclic graph used for covariate selection in the relationship between greenness and CVD incidence.

**Fig.S2.** Exposure-response curves for greenness and risk of CVD and its subtypes incidence.

**Methods** The formula for statistical significance of differences among subgroup analyses by the calculation of the 95% confidence interval (CI) as:

$Upper limit of 95\% CI = \left( Q2-Q1 \right) + 1.96\sqrt{{SE}_{1}^{2}+{SE}_{2}^{2}}$

$Lower limit of 95\% CI = \left( Q2-Q1 \right) - 1.96\sqrt{{SE}_{1}^{2}+{SE}_{2}^{2}}$ Equation S1

where Q2 and Q1 are the risk estimates for the target group and reference group, and SE_1_ and SE_2_ are the corresponding standard errors.

**Methods** Quantitative assessments on the preventable CVD incidence if met the WHO recommended levels.

1. Data

We used the NDVI_max_ in 2020 at a 250 m × 250 m resolution, the same as the exposure metrics use in the health effects analysis in this study. The land use data were downloaded from the European Space Agency (ESA, https://cds.climate.copernicus.eu/cdsapp#!/dataset/) with a resolution of 300 m × 300 m in 2020. The population data was downloaded from WorldPop (<https://www.worldpop.org/>) in 2020 at a 1000 m × 1000 m resolution. Above all, we resampled these data to the same resolution based on the NDVI_max_ layer.

(2) Methods

As the WHO recommendation of at least 0.5 hectares within a linear distance of 300 m for universal available green space, studies revealed that an approximate green space coverage of 25% green area (GA) was necessary per unit area. Thus, at the first stage, we calculated the corresponding WHO recommended NDVI levels (NDVI_goal_) at regional level based on the universal WHO recommendation (25%GA) per grid cell (the minimum assessment unit) by the following steps: 1) we conducted a 300-m buffer around each grid centroid of NDVI_max_ in 2020. Then we estimated the mean NDVI_max_ value and the percentage of green space area (%GA) within this buffer area, respectively. 2) we developed a generalized additive model (GAM) to fit the association on %GA- NDVI_max_ for each region in CKB following previous studies. 3) we predicted the NDVI_goal_ if the NDVI levels achieved the WHO recommended scenario (25%GA) per region in CKB.

At the second stage, we evaluated the current population exposure level to NDVI by calculating population-weighted NDVI_max_ (*NDVI_Pop_*) per region in CKB, and compared the current NDVI level to NDVI_goal_ to ascertain whether the region had fulfilled the WHO’s recommendation (Equation S2). If the *NDVI_Pop_* did not achieve NDVI_goal_, we continue to the third stage to calculate preventable proportion of CVD incidence assuming current *NDVI_Pop_* could achieve NDVI_goal._

*NDVI_Pop_ = ∑Pop_i_ × NDVI_i_ / ∑Pop_i_*  Equation S2

where *NDVI_Pop_* represents the population-weighted NDVI_max_ level per CKB region, *Pop_i_* represents the population of grid *i*, and *NDVI_i_* represents the NDVI_max_ value of grid *i* within the CKB region*.*

At the third stage, we assessed the preventable proportion of CVD incidence assuming that current *NDVI_Pop_* could achieve NDVI_goal_ per region. Effect estimate on greenness-CVD incidence was HR value of 0.976 (95% CI: 0.958, 0.994) developed in this study. First, we calculated the exposure difference between the *NDVI_Pop_* and NDVI_goal_ values per region; second, the relative risk (RR) on CVD incidence regarding exposure difference was calculated (Equation S3); third, preventable proportion of CVD incidence if current NDVI level could achieve NDVI_goal_, represented by population attributable fraction (PAF), was calculated based on *RR*_exposure difference_ following Equation S4.

$RR\text{exposure difference} = exp[(In HR/exposure\text{increment}) \times exposure difference]$ Equation S3

where *RR*_exposure difference_ is the relative risk on CVD incidence with exposure difference per region; *HR* is the effect estimate on greenness-CVD incidence within a 500 m buffer in our study; *exposure*_increment_ is the unit increment of NDVI to calculate HR, which is 0.1 in this study; and *exposure difference* indicates the greenness difference between the current population-weighted NDVI_max_ (*NDVI_Pop_*) and the WHO recommended NDVI_max_ values (NDVI_goal_) per city.

$PAF = [(RR\text{exposure difference}\text{ }-1) / RR\text{exposure difference}]$ Equation S4

where *PAF* is the proportional decreasing in CVD incidence if greenness increased to the WHO recommended exposure level per region.

**Table S1.** SES and health characteristics of participants in China Kadoorie Biobank at baseline, stratified by subgroups of MET

| **Variables** | **MET: Low** | **MET: Middle** | **MET: High**  (n=164,797) | *P* value |  |
| --- | --- | --- | --- | --- | --- |
|  | (n = 180,811) | (n = 167,083) |  |  |  |
| **Income** (n, %) |  |  |  |  |  |
| <19,999 yuan/year | 112,593 (62.3) | 91,396 (54.7) | 89,678 (54.4) | <0.001 |  |
| ≥20,000 yuan/year | 68,218 (37.7) | 75,687 (45.3) | 75,119 (45.6) |  |  |
| **Education** (n, %) |  |  |  |  |  |
| No formal education | 32,267 (17.8) | 28,180 (16.9) | 34,723 (21.1) | <0.001 |  |
| Primary school | 61,830 (34.2) | 51,548 (30.9) | 51,795 (31.4) |  |  |
| High school | 76,539 (42.3) | 71,926 (43.0) | 73,910 (44.8) |  |  |
| College or higher | 10,175 (5.6) | 15,429 (9.2) | 4369 (2.7) |  |  |
| **BMI** (n, %) |  |  |  |  |  |
| <18.5 kg/m^2^ | 8864 (4.9) | 7161 (4.3) | 6335 (3.8) | <0.001 |  |
| 18.5–24.9 kg/m^2^ | 103,848 (57.4) | 105,734 (63.3) | 111,841 (67.9) |  |  |
| ≥25 kg/m^2^ | 68,098 (37.7) | 54,187 (32.4) | 46,621 (28.3) |  |  |
| **Self-rated health** (n, %) |  |  |  |  |  |
| Excellent | 25,893 (14.3) | 32,666 (19.6) | 31,833 (19.3) | <0.001 |  |
| Good | 42,889 (23.7) | 46,788 (28.0) | 54,526 (33.1) |  |  |
| Fair | 87,351 (48.3) | 71,835 (43.0) | 65,828 (39.9) |  |  |
| Poor | 24,678 (13.6) | 15,794 (9.5) | 12,610 (7.7) |  |  |

BMI, body mass index; MET, metabolic equivalent of task, low: <13 hours/day, middle: 13−25.9 hours/day, high: ≥26 hours/day.

**Table S2.** Hazard ratios (HRs) and 95% confidence intervals (95% CI) of CVD incidence per 0.1 increment in NDVI_ann-mean_ within 500 m and 1000 m buffers

| **Outcome** | **500 m buffer** | **1000 m buffer** |
| --- | --- | --- |
| Cardiovascular disease (CVD) | 0.959 (0.931, 0.987) | 0.946 (0.918, 0.975) |
| Ischaemic heart disease (IHD) | 0.930 (0.899, 0.962) | 0.921 (0.888, 0.955) |
| Acute myocardial infarction (AMI) | 0.873 (0.820, 0.930) | 0.869 (0.815, 0.926) |
| Total Stroke | 0.930 (0.906, 0.954) | 0.925 (0.898, 0.953) |
| Hemorrhagic stroke (HS) | 0.999 (0.965, 1.035) | 1.007 (0.970, 1.046) |
| Ischaemic stroke (IS) | 0.917 (0.890, 0.944) | 0.912 (0.883, 0.942) |

NDVI_ann_mean_, the overall average level of NDVI_annual_; This model included strata of regions and clusters of clinics, and adjustment for age, sex, smoking, secondhand smoke, alcohol drinking, education, income, BMI, MET, fresh fruits, fresh vegetables, fish, meat, cooking fuels, heating fuels and self-rated health.

| **Outcome** | **500 m buffer** | **1000 m buffer** |
| --- | --- | --- |
| Cardiovascular disease (CVD) | 0.975 (0.957, 0.993) | 0.964 (0.945, 0.983) |
| Ischaemic heart disease (IHD) | 0.957 (0.935, 0.980) | 0.953 (0.929, 0.978) |
| Acute myocardial infarction (AMI) | 0.930 (0.891, 0.971) | 0.923 (0.884, 0.964) |
| Total Stroke | 0.951 (0.933, 0.970) | 0.948 (0.926, 0.970) |
| Hemorrhagic stroke (HS) | 0.998 (0.972, 1.025) | 1.002 (0.974, 1.032) |
| Ischaemic stroke (IS) | 0.941 (0.921, 0.962) | 0.937 (0.913, 0.961) |

**Table S3.** Hazard ratios (HRs) and 95% confidence intervals (95% CI) of CVD incidence per 0.1 increment in NDVI_max_ within 500 m and 1000 m buffers by excluding the participants with self-reported CVD at baseline

This model included strata of regions and clusters of clinics, and adjustment for age, sex, smoking, secondhand smoke, alcohol drinking, education, income, BMI, MET, fresh fruits, fresh vegetables, fish, meat, cooking fuels, heating fuels and self-rated health.

**Table S4.** Hazard ratios (HRs) and 95% confidence intervals (95% CI) of CVD incidence per 0.1 increment in NDVI_max_ within 500 m and 1000 m buffers by excluding the participants with follow-up time less than 2 years

| **Outcomes** | **500 m buffer** | **1000 m buffer** |
| --- | --- | --- |
| Cardiovascular disease (CVD) | 0.987 (0.969, 1.006) | 0.978 (0.959, 0.998) |
| Ischaemic heart disease (IHD) | 0.962 (0.942, 0.983) | 0.955 (0.932, 0.979) |
| Acute myocardial infarction (AMI) | 0.943 (0.900, 0.987) | 0.939 (0.895, 0.984) |
| Total Stroke | 0.954 (0.934, 0.973) | 0.951 (0.928, 0.975) |
| Hemorrhagic stroke (HS) | 1.002 (0.976, 1.028) | 1.009 (0.982, 1.037) |
| Ischaemic stroke (IS) | 0.944 (0.923, 0.966) | 0.941 (0.916, 0.967) |

This model included strata of regions and clusters of clinics, and adjustment for age, sex, smoking, secondhand smoke, alcohol drinking, education, income, BMI, MET, fresh fruits, fresh vegetables, fish, meat, cooking fuels, heating fuels and self-rated health.

**Table S5.** Hazard ratios (HRs) and 95% confidence intervals (95% CI) of CVD incidence per 0.1 increment in NDVI_max_ within 500 m and 1000 m buffers for the redefinition of the subgroups of BMI

| **Outcomes** | **500 m buffer** | **1000 m buffer** |
| --- | --- | --- |
| Cardiovascular disease (CVD) | 0.979 (0.961, 0.997) | 0.969 (0.950, 0.989) |
| Ischaemic heart disease (IHD) | 0.958 (0.935, 0.981) | 0.953 (0.928, 0.979) |
| Acute myocardial infarction (AMI) | 0.938 (0.898, 0.980) | 0.934 (0.893, 0.976) |
| Total Stroke | 0.952 (0.934, 0.971) | 0.949 (0.928, 0.971) |
| Hemorrhagic stroke (HS) | 1.004 (0.979, 1.029) | 1.009 (0.982, 1.037) |
| Ischaemic stroke (IS) | 0.942 (0.923, 0.963) | 0.939 (0.915, 0.963) |

This model included strata of regions and clusters of clinics, and adjustment for age, sex, smoking, secondhand smoke, alcohol drinking, education, income, BMI, MET, fresh fruits, fresh vegetables, fish, meat, cooking fuels, heating fuels and self-rated health. The redefinition of the subgroups of BMI included underweight (<18.5 kg/m^2^), normal weight (18.5−23.9 kg/m^2^), and overweight/obesity (≥24 kg/m^2^).

**Table S6.** Hazard ratios (HRs) and 95% confidence intervals (95% CI) of CVD incidence per 0.1 increment in NDVI_max_ within 500 m and 1000 m buffers after further adjustment of PM_2.5_ and O_3_

| **Outcomes** | **Confounders** | **500 m buffer** | **1000 m buffer** |  |
| --- | --- | --- | --- | --- |
| Cardiovascular disease (CVD) | PM_2.5_ | 0.977 (0.959, 0.996) | 0.967 (0.948, 0.986) | |
|  | O_3_ | 0.976 (0.958, 0.994) | 0.966 (0.947, 0.985) | |
| Ischaemic heart disease (IHD) | PM_2.5_ | 0.955 (0.933, 0.978) | 0.950 (0.925, 0.976) | |
|  | O_3_ | 0.957 (0.934, 0.980) | 0.952 (0.927, 0.977) | |
| Acute myocardial infarction (AMI) | PM_2.5_ | 0.939 (0.899, 0.982) | 0.935 (0.894, 0.978) | |
|  | O_3_ | 0.935 (0.895, 0.977) | 0.931 (0.890, 0.973) | |
| Total Stroke | PM_2.5_ | 0.952 (0.934, 0.969) | 0.949 (0.928, 0.969) | |
|  | O_3_ | 0.951 (0.933, 0.969) | 0.948 (0.927, 0.969) | |
| Hemorrhagic stroke (HS) | PM_2.5_ | 1.004 (0.979, 1.030) | 1.010 (0.982, 1.038) | |
|  | O_3_ | 1.001 (0.976, 1.026) | 1.006 (0.979, 1.033) | |
| Ischaemic stroke (IS) | PM_2.5_ | 0.942 (0.923, 0.961) | 0.938 (0.915, 0.960) | |
|  | O_3_ | 0.941 (0.922, 0.961) | 0.937 (0.914, 0.961) | |

This model included strata of regions and clusters of clinics, and adjustment for age, sex, smoking, secondhand smoke, alcohol drinking, education, income, BMI, MET, fresh fruits, fresh vegetables, fish, meat, cooking fuels, heating fuels, self-rated health, PM_2.5_ or O_3_.

**Table S7.** The proportion of mediation of associations between greenness and CVD incidence within a 500 m buffer, by air pollutants

| Outcome | PM_2.5_ | | O_3_ | |
| --- | --- | --- | --- | --- |
|  | Prop. mediated (95% CI) | *P* | Prop. mediated (95% CI) | *P* |
| Cardiovascular disease (CVD) | 0.003 (-0.002, 0.008) | 0.28 | 0.528 (0.462, 0.594) | <0.001 |
| Ischaemic heart disease (IHD) | 0.011 (0.004, 0.018) | 0.001 | 0.458 (0.377, 0.539) | <0.001 |
| Acute myocardial infarction (AMI) | -0.019 (-0.041, 0.002) | 0.08 | 0.131 (0.033, 0.229) | 0.01 |
| Total Stroke | -0.005 (-0.008, -0.002) | 0.002 | 0.264 (0.232, 0.295) | <0.001 |
| Ischaemic stroke (IS) | -0.001 (-0.003, 0.001) | 0.25 | 0.209 (0.186, 0.233) | <0.001 |

PM_2.5_, fine particulate matter; O_3_, ozone; Covariates were adjusted including age, sex, smoking, secondhand smoke, alcohol drinking, education, income, BMI, MET, fresh fruits, fresh vegetables, fish, meat, cooking fuels, heating fuels and self-rated health.


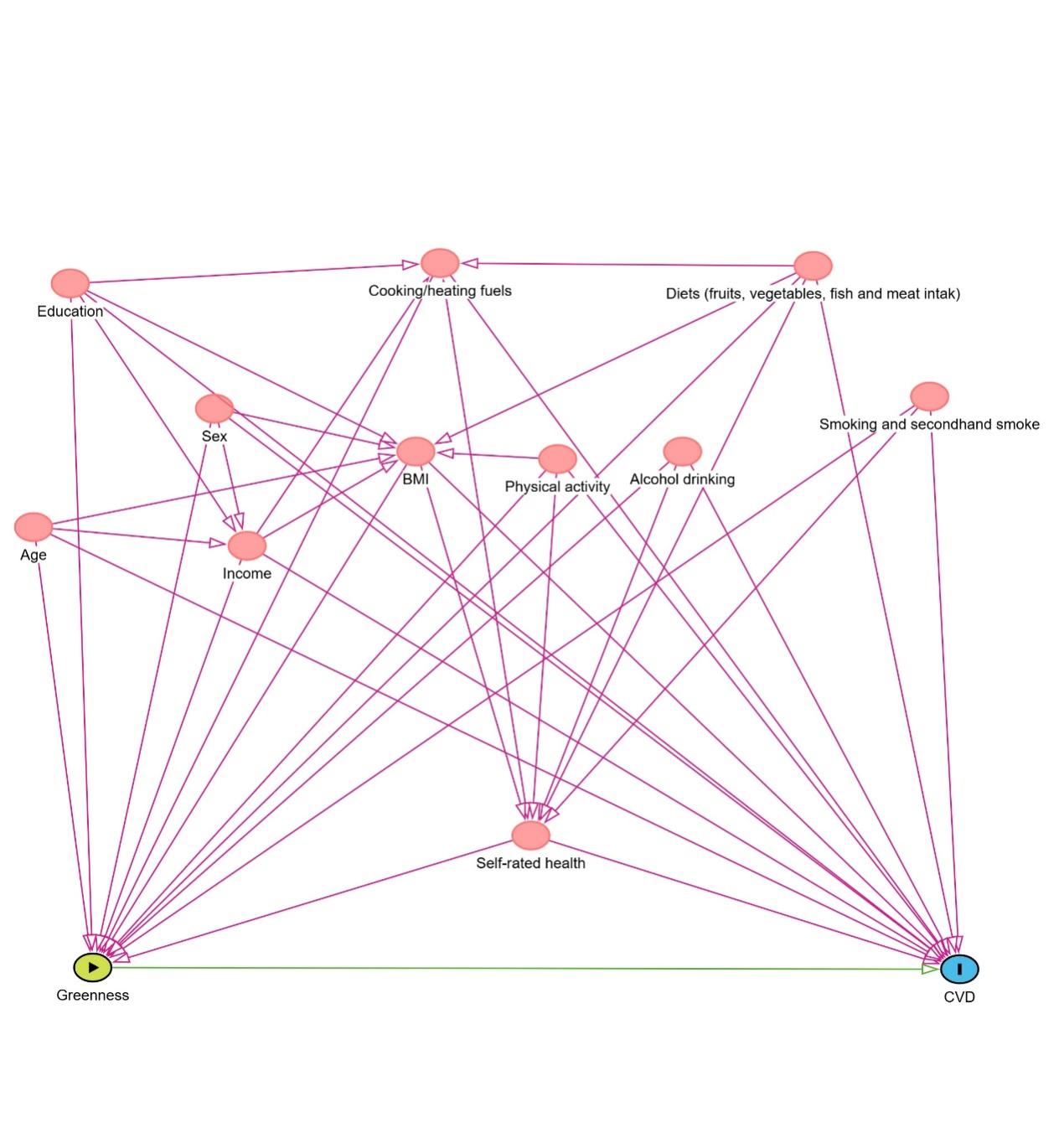


**Fig. S1.** Directed acyclic graph used for covariate selection in the relationship between greenness and CVD incidence.

**Fig. S2.** Exposure-response curves for greenness and risk of CVD and its subtypes incidence. Covariates were adjusted as the fully adjusted model including age, sex, smoking, secondhand smoke, alcohol drinking, education, income, BMI, MET, fresh fruits, fresh vegetables, fish, meat, cooking fuels, heating fuels and self-rated health.


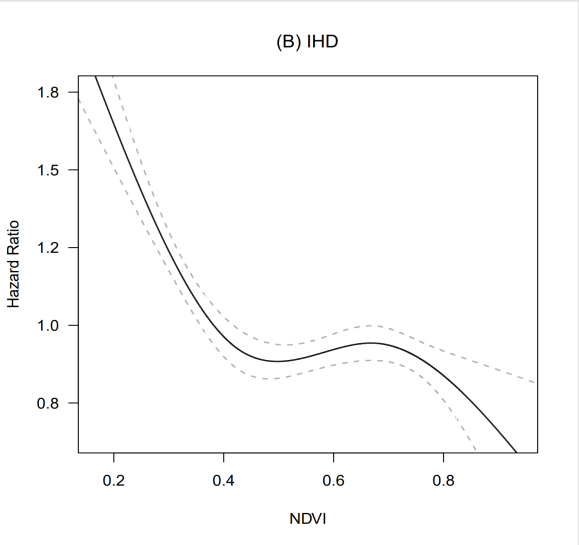

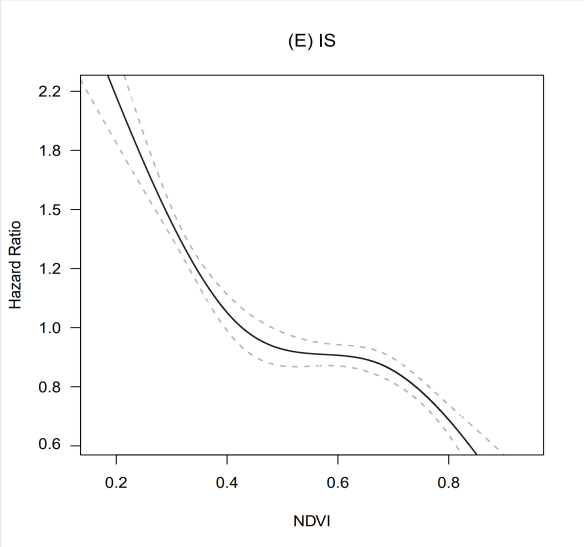

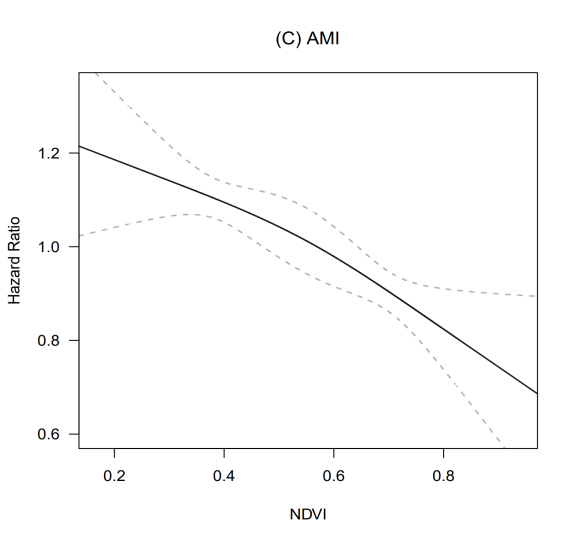

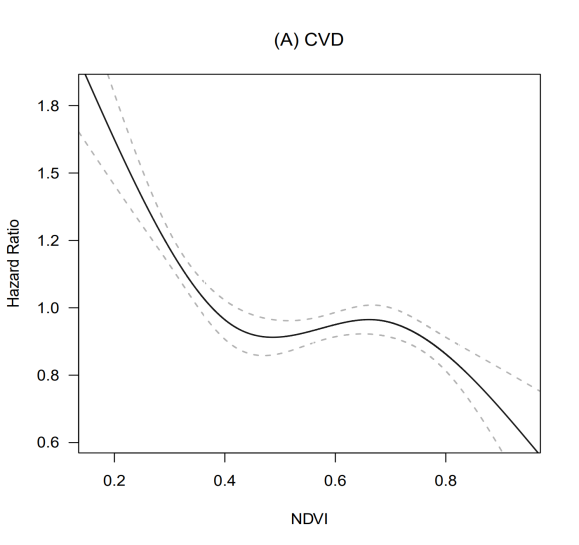

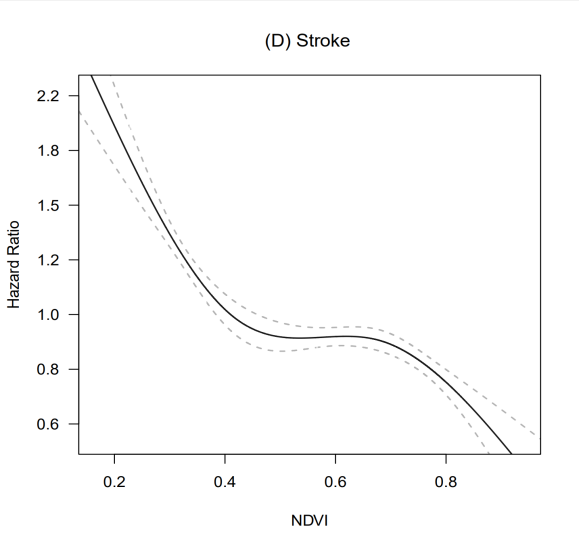

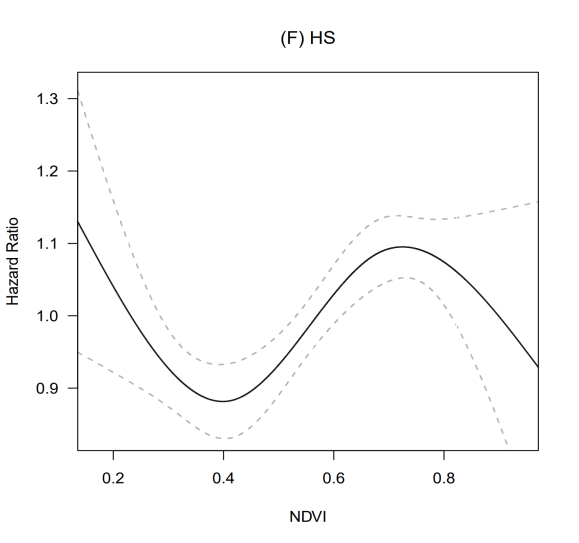

Supplement: Multimedia component 1 [file mmc1.docx]
